# Effects of Heat Acclimation on Photosynthesis, Antioxidant Enzyme Activities, and Gene Expression in Orchardgrass under Heat Stress

**DOI:** 10.3390/molecules190913564

**Published:** 2014-09-01

**Authors:** Xin Xin Zhao, Lin Kai Huang, Xin Quan Zhang, Zhou Li, Yan Peng

**Affiliations:** Grassland Science Department, Sichuan Agricultural University, Ya’an 625014, China; E-Mails: zxx19890727@163.com (X.X.Z); huanglinkai@sicau.edu.cn (L.K.H.); lizhou1234567890@yeah.net (Z.L.); pengyanlee@163.com (Y.P.)

**Keywords:** heat acclimation, antioxidative enzyme, gene expression, photosynthesis, orchardgrass (*Dactylis glomerata* L.)

## Abstract

The present study was designed to examine the effects of heat acclimation on enzymatic activity, transcription levels, the photosynthesis processes associated with thermostability in orchardgrass (*Dactylis glomerata* L.).The stomatal conductance (Gs), net photosynthetic rate (Pn), and transpiration rates (Tr) of both heat-acclimated (HA) and non-acclimated (NA) plants were drastically reduced during heat treatment [using a 5-day heat stress treatment (38/30 °C ‒ day/night) followed by a 3-day recovery under control conditions (25/20 °C ‒ day/night), in order to consolidate the second cycle was permitted]. Water use efficiency increased more steeply in the HA (4.9 times) *versus* the NA (1.8 times) plants, and the intercellular CO_2 _ concentration decreased gently in NA (10.9%) and HA (25.3%) plants after 20 d of treatments compared to 0 days’. Furthermore, heat-acclimated plants were able to maintain significant activity levels of superoxide disumutase (SOD), catalase (CAT), guaiacol peroxidase (POD), and transcription levels of genes encoding these enzymes; in addition, HA plants displayed lower malondialdehyde content and lower electrolyte leakage than NA plants. These results suggest that maintenance of activity and transcription levels of antioxidant enzymes as well as photosynthesis are associated with variable thermostability in HA and NA plants. This likely occurs through cellular membrane stabilization and improvements in water use efficiency in the photosynthetic process during heat stress. The association between antioxidant enzyme activity and gene expression, both of which may vary with genetic variation in heat tolerance, is important to further understand the molecular mechanisms that contribute to heat tolerance.

## 1. Introduction

The mean annual global surface temperature is expected to increase between 1.1 °C to 6.4 °C by the end of the 21st century [1]. The negative impacts of climate trends on the yields of crops and forages have already occurred on a global scale [2]. Heat stress is one of the primary abiotic stressors that may lead to dramatic reductions in the economic yield of crop plants [3,4] and limit the growth of cool-season plant species in many regions of the world.

An increased capacity for thermotolerance and delayed thermal injury contribute to the heat tolerance of plants [5]. Studies have shown that protection from heat-stress injury can be induced in heat-sensitive, cool-season plants by subjecting these plants to a heat-acclimation pretreatment; this was found to be partially due to the induction of antioxidative compounds, which helped to prevent the accumulation of reactive oxidant species (ROS) and membrane lipid peroxidation during heat stress [6]. Heat stress alters the physiological, biochemical, and molecular responses of plants. Photosynthetic activity is an important temperature-dependent functional trait that is linked to plant metabolism and performance [7]. Studies have also shown that the photosynthetic responses to heat stress demonstrated synergistic trends implicating internal CO_2_ concentration, net photosynthesis ,and stomatal conductance; the modifications of net photosynthesis were also associated with changes in stomatal control [8,9,10].

Furthermore, plants can acclimate to high temperature-induced oxidative stress by increasing the expression of genes involved in antioxidant systems. Plants have evolved an antioxidant defense system consisting of both nonenzymatic and enzymatic constituents in plant cells, which minimizes or even eliminates oxidative damage [11,12]. The study of gene expression underlying the changes in antioxidant enzyme activities could provide insight into the molecular adaptation of plants to heat-stress conditions [13]. Examining the effects of heat-stress on the expression of genes coding for the key enzymes superoxide dismutase (SOD), Peroxidase (POD), and catalase (CAT) could provide such insight.

Orchardgrass (*Dactylis glomerata* L.) is a cool-season, perennial forage grass, considered the fourth most important forage grass in the temperate areas of the world [14]. The effects of heat acclimation on subsequent heat tolerance mechanisms are not well understood in orchardgrass. For example, antioxidant metabolisms may respond differently to heat-stress in different orchardgrass varieties, and how antioxidant enzyme activities and the expression of gene encoding antioxidant enzymes are affected by heat-stress conditions are not well understood. Moreover, it could be important to know whether antioxidant enzyme activities are associated with better photosynthetic ability and whether heat acclimation plays a significant role in heat resistance in orchardgrass. Here, we investigated changes in photosynthetic activity, antioxidant enzyme activities, and the expression patterns of genes encoding antioxidant enzymes in leaves of orchardgrass under heat stress, and compared these responses in NA and HA samples. We aimed to see whether antioxidant system plays an important role in enhanced heat tolerance, performance of orchardgrass after heat acclimation, and to investigate the relationship between antioxidant enzyme activities and their correspondent gene expressions. We expected to solve the problem that whether the impacts on antioxidant enzyme activities of heat acclimation are controlled by their correspondent gene regulation. Insights from this study will help us further understand the interactive mechanisms of plant thermal tolerance and heat acclimation, as well as inform the selection of heat-tolerant varieties, thus improving cultivation in a changing climate.

## 2. Results

### 2.1. Physiological Responses to Heat Stress Following Heat Preconditioning and Non-Preconditioning

**Figure 1:**
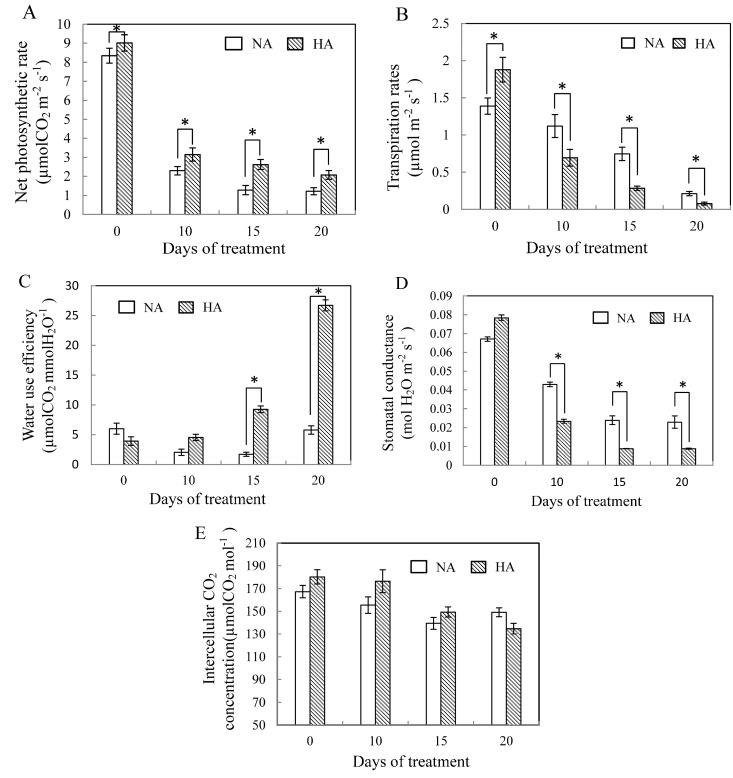
Changes in the amount of net photosynthetic rate (**A**), transpiration rates (**B**), water use efficiency (**C**), stomatal conductance (**D**) and intercellular CO_2_ concentration (**E**) for NA and HA under heat stress for 20 days. Columns marked with asterisks indicate significant difference between measurements made at 0, 10, 15, and 20 day of treatment for a given treatments based on a least significant difference test (*p* ≤ 0.05). Data are provided as mean ± SE of four independent measurements.

In our experiment, the net photosynthetic rate and transpiration rate were decreased significantly after a prolonged heat stress period (Figure 1A,B). The reduction in net photosynthetic rate was lower, while the transpiration rate was higher in HA plants than in NA plants. The net photosynthetic rate decreased to almost the same level during treatment, and the transpiration rates between HA and NA plants tended to show similar changes in net photosynthetic rate under heat stress (Figure 1B). Both HA and NA plants experienced an increasing water use efficiency (WUE) in response to heat stress, however, the WUE was significantly higher in HA plants than in NA plants (*p* ≤ 0.05). WUE increased more steeply in HA plants (4.9 times) than in NA plants (1.8 times) after 20 days under heat stress and this ascent was quicker in HA plants than in NA plants (Figure 1C). The differences in WUE between NA and HA plants were not significant at 0 day or 10 day. There was a significant difference and a substantial decrease in stomatal conductance between NA and HA plants under heat stress over the course of the entire period, and the HA plants was a little higher than NA plants at the beginning (Figure 1D).

**Figure 2:**
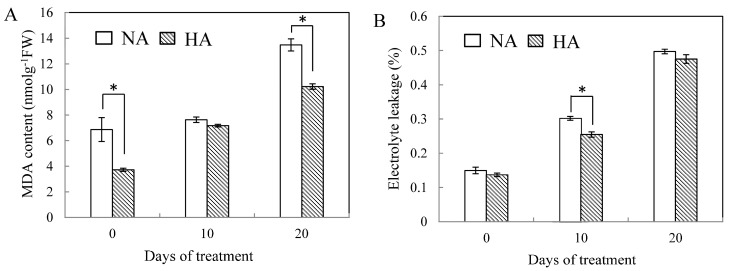
Effects of heat stress on the content of MDA (**A**) and electrolyte leakage (EL) (**B**) in leaves of both heat-acclimated (HA) and non-acclimated (NA) plants. Error bars represent standard error of the mean. Asterisks above columns indicate a significant treatment difference between HA and NA, LSD (*p* ≤ 0.05).

Intercellular CO_2_ concentration progressively declined in NA plants (10.9%) and HA plants (25.3%) during the entire treatment, and there is no difference between NA and HA plants during the experiment (*p* ≤ 0.05). Cellular membrane stability was estimated by examining EL and MDA content in the leaves. As the number of days of heat stress increased, MDA content increased regardless of whether or not the plant had undergone heat acclimation. MDA content increased by 90% from the baseline score (day 0) and 1.7-fold in NA and HA plants (Figure 2A). The differences in MDA content among the two treatments were significantly different at 0 day and 20 day under heat stress (Figure 2A).

Heat stress caused a significantly increasing amount of EL as the days increased at 38/30 °C (day/night); this increase was also seen in both HA and NA plants. However, HA plants had lower EL levels than NA plants at 0 d and 20 d under heat stress (Figure 2B).

### 2.2. Effects of Preconditioning and Non-preconditioning on Antioxidant Enzyme Activity Under Heat Stress

**Figure 3:**
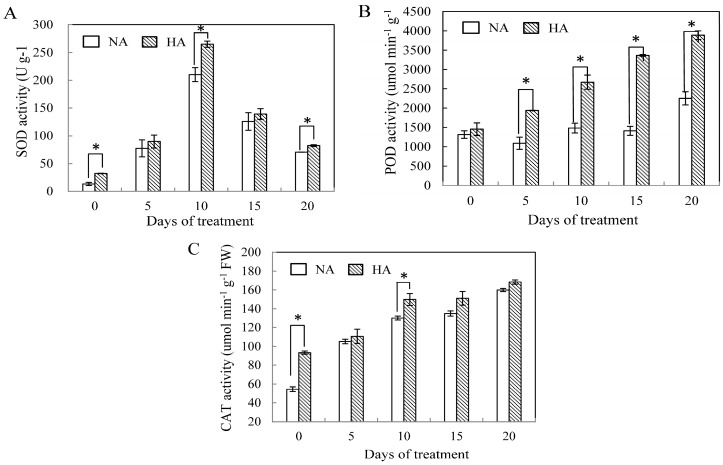
Antioxidant enzyme activities of SOD (**A**), POD (**B**) and CAT (**C**) in leaves of heat-acclimated (HA) and non-acclimated (NA) plants as affected by high temperature. Error bars represent standard error of the mean. Asterisks above columns indicate a significant treatment differences between NA and HA. LSD (*p* ≤ 0.05).

In this study, all of the enzymes exhibited significant changes in activity under stressful conditions. Specifically, there is a pronounced and rapid increase in Cu/ZnSOD activities at 10 day under heat stress for both HA and NA plants and this activity then slightly decreased after 10 days (Figure 3A). HA plants maintained higher Cu/ZnSOD activities than NA plants over the course of the 20 days period under heat stress, and showed significantly higher than NA plants at 0 day, 10 day and 20 day (*p* ≤ 0.05).

POD activity increased significantly during the entire treatment period in both HA and NA plants (Figure 3B). The POD of HA plants showed significant activities (*p* ≤ 0.05) compared with NA plants under heat treatment, and these levels were higher by 72.6% than levels detected in NA plants at 20 day. CAT activity levels were slightly increased during the treatment in both HA and NA plants (Figure 3C). However, there is a significant increasing in CAT activity only at 0 day and 10 day (*p* ≤ 0.05).

### 2.3. Assessment of Antioxidant Enzyme Gene Expression under Heat Stress

**Figure 4:**
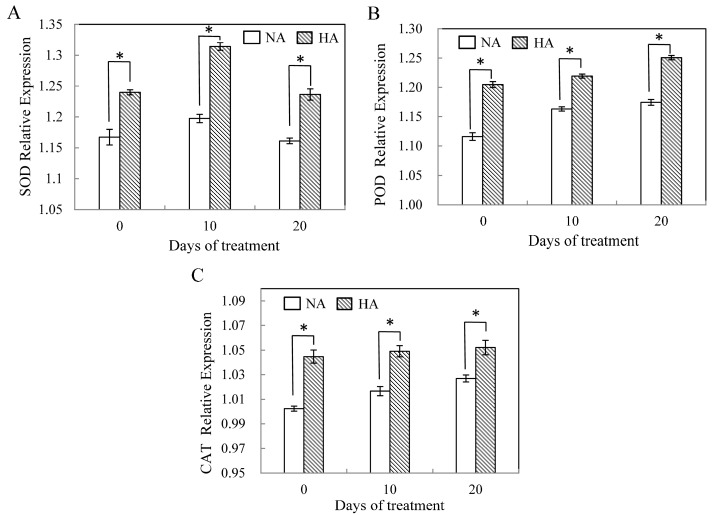
The relative expression level of SOD (**A**), POD (**B**) and CAT (**C**) in leaves of heat-acclimated (HA) and non-acclimated (NA) plants as affected by high temperature. Error bars represent standard error of the mean. Asterisks above columns indicate a significant treatment differences between HA and NA. LSD (*p* ≤ 0.05).

In this study, the transcript abundance of all the genes encoding antioxidant enzymes increased with increasing number of days under heat treatment in HA plants. Corresponding changes in NA plants were relatively small. The expression levels of Cu/ZnSOD in both HA and NA plants strongly increased under heat stress, and then decreased to their respective baseline levels (0 day) after 20 days (Figure 4A). The response of POD activity to stress was different from SOD activity. A continuous increase in POD activity was observed in both HA and NA plants during the period of 20 days. The expression of POD-encoding genes slowly increased but was significant in both HA and NA plants during treatment (Figure 4B). Expression of CAT-encoding genes was up-regulated in the leaves under heat stress. Additionally, there are similar patterns of CAT gene expression in both HA and NA plants.

## 3. Discussion

One major impact of heat stress is cellular membrane damage caused by oxidative stress. It has been found that various environmental stressors (including heat stress) cause oxidative stress resulting from the accumulation of ROS [15]. It has been suggested that a decrease in cell membrane stability reflects high levels of lipid peroxidation caused by ROS [16]. Because of this, the accumulation of MDA is used as an indicator of lipid peroxidation. Maintenance of membrane integrity is a major mechanism of heat tolerance in a number of plant species [3], and it is known that thermotolerance might be acquired in some species through heat acclimation; this can occur through exposure to a non-lethal heat treatment (either reduced temperature or duration of exposure) [17]. Heat acclimation might be helpful in alleviating the membrane lipid peroxidation of plants under heat stress [18]. Our results also showed that increases in both EL and MDA content during prolonged heat stress indicate that cell membranes were injured under heat stress and that NA plants were damaged more severely compared with HA plants. Less MDA content accumulated under heat stress of HA versus NA plants suggests that lower levels of lipid peroxidation occurred because of the increased Cu/ZnSOD levels, higher CAT levels, and higher POD activity; and lower EL found in HA plants. These results indicate that membrane lipid peroxidation occurred due to malfunction of the scavenging system, which may have led to damage in the main cellular components of the plants [19].

Abiotic stresses are caused by the enhanced production of ROS, which further damage the photosynthetic machinery [20]. Photosynthesis is, like all other physiological processes, temperature dependent [21]. During heat acclimation, the threshold temperature is the temperature at which fluidity still maintains the native membrane structure and function [22]. We frequently observed a marked decrease in the net photosynthetic rate and the transpiration rates during high temperature stress (Figure 2A,B). We observed that WUE also significantly increased due to an increase in the net photosynthetic and transpiration rates. WUE increases during exposure to high temperature stress because of the large amount of ROS, which induce inhibition of photosynthesis and damages to the mechanisms of photosynthesis [23]. The WUE of heat-acclimation pretreatment was higher than the non-preconditioned plants, indicating that heat acclimation allowed plants to become more efficient responders to heat stress, HA plants can survive the heat stress better than NA plants. This result may also suggest that HA plants were able to maintain a balance between photosynthesis and transpiration, which could help them survive heat stress through more efficient use of water. This would occur without the loss of photosynthetic capacity. However, the stomatal aperture appears to be controlled by complex mechanisms that operate to maintain a balance between allowing CO_2_ uptake to proceed, restricting the loss of water vapor and preventing leaf desiccation [24]. We observed a decline in stomatal conductance, and intercellular CO_2_ concentration also slowly decreased during the course of our study. However, the decreased Pn we observed may not have been due to increased stomatal limitation, since we also observed a decreased Ci. Our observations were consistent with a previous study in two citrus species under high temperatures (38 °C) [25]. In general, high temperature stress has a direct thermodynamic effect that suppresses biological processes, including the activity of Rubisco enzyme and PS2 [26,27]. In this study, Gs significantly decreased in both HA and NA plants during heat stress, while intercellular CO_2_ concentration did not show similar changes with the decrease of Gs under heat stress. Furthermore, HA plants maintained significantly higher net photosynthetic rate in comparison with control plant under heat stress, but intercellular CO_2_ concentration did not show significant difference between HA and NA treatment. These results indicated that limit photosynthesis of the orchardgrass was caused by non-stomatal factors during heat stress.

Heat acclimation can improve the ability of a plant to adapt adverse environmental conditions, which may result from changes in many physiological and biochemical responses in plants [6]. Several studies have suggested that differences in abiotic stress tolerance may be partially due to the higher constitutive antioxidant enzyme activities in tolerant versus intolerant species [6,23,28]. Therefore, the antioxidant defense mechanism is part of heat stress adaptation and is associated with the acquisition of thermotolerance. Our results support this conclusion, with HA plants having higher Cu/ZnSOD, POD, and CAT antioxidant enzymes activities versus what we observed in NA plants. Obviously, the activity of Cu/ZnSOD, POD, and CAT showed significant differences from baseline (day 0) in HA plants during heat stress, indicating that HA plants’ ability to scavenge H_2_O_2_ strongly improved following pretreatment. Previous research has shown that gene expression underlying changes in antioxidant enzyme activities may facilitate molecular adaptation of plants, and that the maintenance of higher levels of antioxidant enzyme transcripts could play a critical role in protecting plants from oxidative stress [18]. Interestingly, the transcript levels of SOD in our heat acclimated pretreatment were strongly enhanced by heat stress (10 day), and then decreased after 10 days of treatment (Figure 4A). Under the same conditions, genes encoding both POD and CAT enzymes had similar changes to Cu/ZnSOD. Higher levels of Cu/ZnSOD and POD transcripts (Figure 4A,B) in heat-acclimated pretreatment plants suggests that Cu/ZnSOD and POD enzymes could contribute to scavenging for reactive oxidation species during oxidative injury induced by such ROS. Our results show that the gene expression patterns of Cu/ZnSOD, CAT and POD encoding genes were similar, suggestive of similar enzyme activity levels as well. Indeed, CAT transcript and enzyme activities both increased in the leaves of wheat under abiotic stress [29]. Our study demonstrated that enzyme activities and the expression of antioxidant enzyme genes play important roles in the heat tolerance of plants. A previous study showed that both the activity and relative expression levels of antioxidant enzymes did not occur concurrently [30], and that no protection from stress was conferred by such enzymes [31]. Our results proved that the relationship between antioxidant enzyme activities and their correspondent gene expressions might be a positive correlation. However, these differences could be attributed to the complexity of the ROS detoxification system. For example, the changes in gene expression that we observed may not be caused by mRNA levels but could instead be regulated at the posttranscriptional level. In addition, changing activity of one enzyme may not impact the capacity of the pathway as a whole.

## 4. Experimental Section

### 4.1. Plant Materials and Treatments

Specimens of the orchardgrass (*Dactylis glomerata* L.), cultivar ‘01998’, were collected from a field in the research farm of Sichuan Agricultural University in Ya’an, China. Plants (2-year-old) were grown in pots (24 cm diameter by 30 cm deep) filled with a mixture of sterilized sand and loamy soil (1:2, v:v). Plants were grown in a greenhouse for a month and then transferred to a controlled growth chamber with a temperature of 25 °C/20 °C (day/night), 14 h photoperiod, 65% relative humidity, and a photosynthetically active radiation of 1,000 µmol m^−2^s^−1^. Grasses were well watered for two weeks to acclimate them to the environment before heat treatments began. Experiments consisted of 16 pots total (eight per set). One set was used as a NA control, while the other was HA and exposed to two heat stress cycles. Based on a preliminary experiment under heat stress (38 °C/30 °C, day/night), there is a subtle change around the fifth day. Therefore, according to [32,33] and our preliminary experiments, we put it into effect with some modifications. Plants were first transferred from the control regimen (25 °C/20 °C, day/night) to the high-temperature regimen (38 °C/30 °C, day/night) until leaf wilting occurred (5 days stress). Plants were then permitted to recover at control conditions for 3 days and then subjected to the second cycle of heat stress described above. After heat preconditioning, HA and NA plants were exposed to heat stress (38 °C/30 °C, day/night) for 20 days in growth chambers. Plants were well watered under both control and heat stress conditions to prevent drought stress.

### 4.2. Photosynthetic Characteristics and Cell Membrane Stability

The net photosynthetic rate (Pn), transpiration rate (Tr), intercellular CO_2_ concentration (Ci) and stomatal conductance (Gs) were measuring using a portable photosynthetic system (LI-6400, Li-Cor, Lincoln, NE, USA). The photosynthetic active radiation (PAR) was 1,200 µmol·m^−2^s^−1^ and the CO_2_ concentration was 400 µmol mol^−1^. Stomatal conductance (Gs) and Ci were determined at a saturated light intensity of 1,000 µmol m^−2^s^−1^, and 70% relative humidity. Thirty leaves were selected randomly in each pot for each measurement. Cellular membrane stability, expressed as electrolyte leakage (EL), was determined following the methods of Blum and Ebercon [34]. All of the above measurements were made at 10:00 in the morning after 0 day, 5 day, 10 day, 15 day and 20 day of heat stress for both HA and NA plants, respectively.

### 4.3. Antioxidant Enzymes and Lipid Peroxidation

For the extraction of antioxidant enzymes, 0.2 g of fresh leaves were randomly sampled from each pot on each sampling date, frozen immediately in liquid nitrogen for 2 min, and chopped and ground in 2 mL solution containing 50 mM phosphate buffer (pH = 7.8) and 1% (w/v) polyvinyl polypyrrolidone. The homogenate was centrifuged at 13,000 rpm for 25 min at 4 °C and the supernatant was collected for use in the antioxidant enzyme activity and malondialdehyde (MDA) production. The membrane lipid peroxidation of the leaves was then measured by examining the amount of MDA in the leaves [35].

The SOD activity was measured by monitoring the rate of p-nitro blue tetrazolium chloride reduction in absorbance at 560 nm [36]. The reaction mixture (1.5 mL) contained 50 mM phosphate buffer (pH = 7.8), 13 mM methionine, 75 µM nitroblue tetrazolium (NBT), 2 µM riboflavin, 0.1 mM EDTA, and 50 µL enzyme extract. POD activity was assayed in a reaction solution containing 1.85 mL 0.1 M HAc-NaAc buffer (pH = 5.0), 1 mL 50% (v/v) guaiacol solution, and 50 µL enzyme extraction by monitoring the increase in absorbance due to guaiacol oxidation at 460 nm [37]. CAT activity was determined following [38], with some modifications. Briefly, we used the disappearance of H_2_O_2_ at 240 nm at 25 °C. The reaction solution contained 50 mM phosphate buffer solution (PBS, pH = 7.0), 15 mM H_2_O_2_, and 50 µL enzyme extract.

### 4.4. Gene Expression

For analysis of the transcriptional levels of cytosolic Cu/ZnSOD, POD and CAT genes, all plant materials were immediately frozen in liquid nitrogen and stored at −70 °C until further experiments. Total RNA was extracted from leaf samples using RNeasy Plant Kit II (Genebase Bioscience, Guangzhou, China) bases on the manufacturer’s instructions. RNA was reverse transcribed using the iScript cDNA Synthesis Kit (Bio-Rad Laboratories Inc., Hercules, CA, USA). The cDNA obtained for each sample was diluted 1:20 with nuclease-free water for qRT-PCR. Orchardgrass expressed sequence tags (ESTs) coding for antioxidant enzyme genes were identified from orchardgrass EST databases. Cytosolic Cu/ZnSOD, POD, CAT and β-actin were identified based on sequence similarity after tblastx analysis with orthologous genes from wheat. Orchardgrass specific β-actin primers served as internal control to normalize cDNA quantity for each treatment sample. Specific primers (Table 1) were used to amplify with the synthesized cDNA for 39 cycles according to the following conditions for each cycle: 95 °C for 10 s, 61.5 °C for 15 s, 72 °C for 15 s, then a final storage at 4 °C. The qRT-PCR analyses were performed on the CFX Connect Real-time PCR detection system (Bio-Rad) using 3 µL of diluted cDNA in a 10 µl reaction volume with 2× IQ SYBR Green Supermix (Bio-Rad). Melting curves were also plotted (60–90 °C) in order to make sure that a single PCR product was amplified for each pair of primers (S.1). The qPCR data were obtained as CT values, and analyzed using the comparative Ct method (2^−ΔΔCt^) [39].

**Table 1:** The primer sequences for real-time qPCR analysis of Cu/ZnSOD, POD, CAT and β-actin genes.

Gene	Species	Accession	Sequence of Primers
**Cu/ZnSOD**	Orchardgrass	HO131072.1	F: 5′-GGCATACTGTCACTCTAAG-3′
			R: 5′- CAACTCCAGGTCATATCG-3′
**CAT**	Orchardgrass	HO179331.1	F: 5′-GCGACGGCATGAAGTTCCC-3′
			R: 5′-CGGTTGACGAGCGTGTAGGTG-3′
**POD**	Orchardgrass	HO125605.1	F: 5′-GCCTCCGTGCTGCTCGAC-3′
			R: 5′-CCAGGATGTCGGAGCAGGAGA-3′
**β-actin**	Orchardgrass	HO135499.1	F: 5′-GTGTTGGATTCTGGTGATGGTGT-3′
			R: 5′-GGCAGTGGTGGTGAAGGAGTAA-3′

### 4.5. Statistical Analysis

In this experiment, samples were randomized, four replicates for two heat treatments, and five sampling dates. Plants were arranged randomly in the growth chambers. To compare the response of HA and NA plants under different treatments, statistical analysis was conducted using an analysis of variance (ANOVA) based on the general linear model. Differences between means were considered significant at least 95% confidence level (*p* ≤ 0.05).

## 5. Conclusions

In conclusion, the present study demonstrated that heat acclimation had an effect on plant thermostability by protecting the membrane system and improving photosynthesis and WUE under heat stress. The different responses of the activities and transcript levels of antioxidant enzymes to heat stress in NA and HA suggested that Cu/ZnSOD, CAT and POD may play important roles in antioxidant protection against damage during heat stress, and heat acclimation pretreatment might have an influence on the gene expression. The effects of heat acclimation on physiological responses are closely related to the expression of genes coding for the key antioxidant enzymes. The results will provide a better understanding of the role of antioxidant enzyme activities and physiological mechanisms of heat acclimation of orchardgrass and subsequently heat tolerance.

## Abbreviations

CAT: Catalase
Ci: Intercellular CO_2_ concentration
EL: Electrolyte leakage
Gs: Stomatal conductance
HA: Heat acclimated
MDA: Malondialdehyde
NA: Non-acclimated
Pn: Net photosynthetic rate
POD: Guaiacol peroxidase
SOD: Superoxide dismutase
Tr: Transpiration rates
WUE: Water use efficiency

## Supplementary Materials

Supplementary materials can be accessed at: http://www.mdpi.com/1420-3049/19/9/13564/s1.

## Supplementary Files

Supplementary File 1
