# Supplementary material for: Effects of Heat Acclimation on Photosynthesis, Antioxidant Enzyme Activities, and Gene Expression in Orchardgrass under Heat Stress
Source: Molecules. 2014 Sep 1;19(9):13564–76. doi: 10.3390/molecules190913564 (PMC6271748; doi:10.3390/molecules190913564)

Supporting Information

Figure S1. Dissociation curves for housekeeping gene (β-actin) (**A**) and antioxidant genes SOD (**B**), CAT (**C**), and POD (**D**) with single peak.

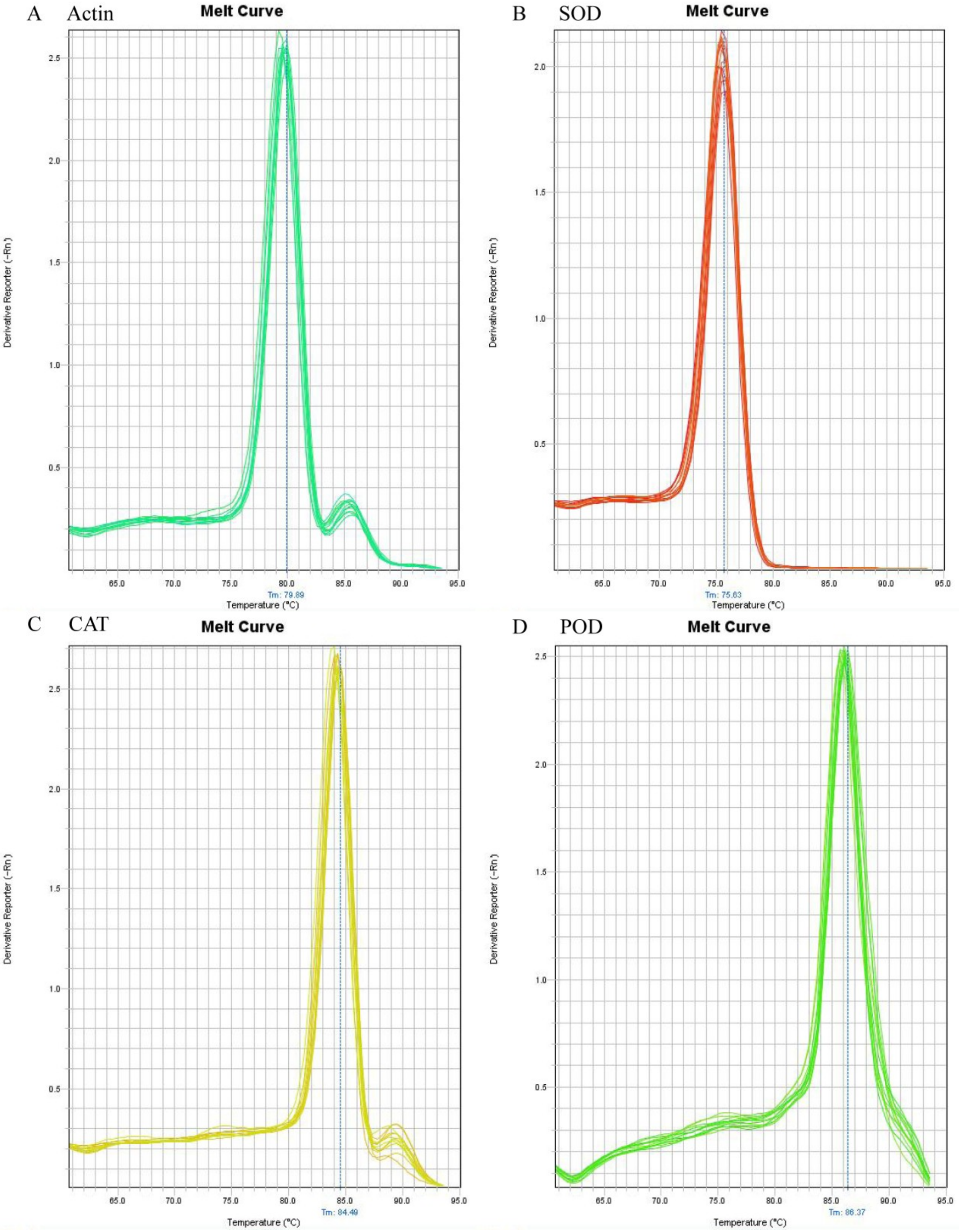

Supplement: Supplementary File 1 [file molecules-19-13564-s001.pdf]
